# Synthetic LGE derived from cardiac T_1 _mapping for simultaneous assessment of focal and diffuse cardiac fibrosis

**DOI:** 10.1186/1532-429X-16-S1-P362

**Published:** 2014-01-16

**Authors:** Kyungpyo Hong, Edward V DiBella, Eugene G Kholmovski, Ravi Ranjan, Christopher J McGann, Daniel Kim

**Affiliations:** 1 UCAIR, Radiology, University of Utah, Salt Lake City, Utah, USA; 2 Division of Cardiology, Internal Medicine, University of Utah, Salt Lake City, Utah, USA

## Background

While late gadolinium enhanced (LGE) MRI is the gold standard for detection of focal myocardial scarring [1], it is less effective than cardiac T_1 _mapping (ECV) for detection of diffuse fibrosis. LGE, in principle, can be synthesized from cardiac T_1 _maps. We sought to derive synthetic LGE images from saturation-recovery based cardiac T_1 _maps for simultaneous assessment of focal and diffuse cardiac fibrosis.

## Methods

We imaged 6 mongrel dogs with lesions created by RF ablation on a 3T MRI system (Verio, Siemens), using arrhythmia-insensitive-rapid (AIR) cardiac T_1 _mapping [2] and standard LGE MRI during equilibrium of Gd-BOPTA (slow infusion at 0.002 mmol/kg/min), in order to compare standard and synthetic LGE images acquired at identical concentration of Gd-BOPTA. Both LGE MRI and cardiac T_1 _mapping were acquired with identical spatial resolution = 1.4×1.4×7 mm. After calculating the AIR cardiac T_1 _maps, as previously described[2], a synthetic LGE image was subsequently synthesized using the Bloch equation describing an ideal inversion recovery: M_z _= 1 - 2*exp(-TI/T_1_), where M_z _is the longitudinal magnetization, inversion time (TI) to null the normal myocardium was calculated by rearranging the above equation as TI = T_1M _× log(2), where T_1M _is the mean T_1 _of normal myocardium. For quantitative analysis, we calculated the contrast ratio, as defined as the signal difference (e.g., lesion-myocardium) divided by lesion (see Table 1). Same analysis was performed for the blood-myocardium pair. This analysis enabled us to compare standard and synthetic LGE data sets with different intensity scales. Pair-wise t-test was used to compare the two groups (standard vs. synthetic LGE).

**Table 1:** Summary of contrast ratio of lesion-myocardium and blood-myocardium pairs.

Tissue Pair	Standard LGE (%)	Synthetic LGE (%)	p-value	Percent Change (%)
Lesion vs. Myocardium	89.8 ± 4.2	96.1 ± 2.2	< 0.001	7.0

Blood vs. Myocardium	88.1 ± 4.8	95.9 ± 2.4	< 0.001	8.9

## Results

**Figure 1:**
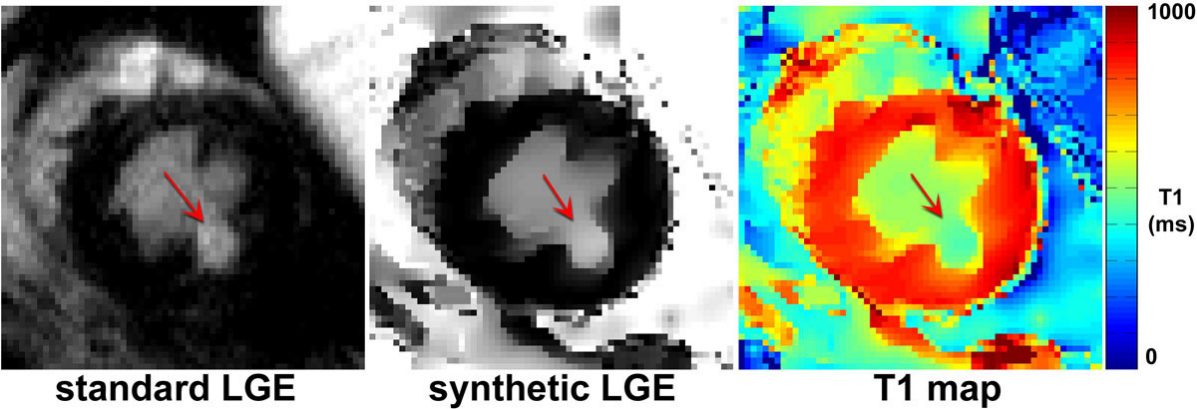
**Comparison (left) standard LGE with (middle) synthetic LGE derived from (right) T_1 _map**. Red arrows point to RF ablation lesion created hours before with a catheter.

Our pooled data contained 21 short-axis planes with different RF lesions. Figure 1 shows representative standard and synthetic LGE images with a lesion. The two LGE images showed comparable image quality. As summarized in Table 1, synthetic LGE yielded higher (p < 0.001) contrast ratio of the lesion-myocardium and blood-myocardium pairs than standard LGE, but the magnitude of the differences was less than 10%.

## Conclusions

We propose a new approach to simultaneously assess focal and diffuse cardiac fibrosis using cardiac T_1 _mapping, with no need for separate acquisition of standard LGE images. This approach is also compatible with inversion-recovery based cardiac T_1 _mapping methods. Synthetic LGE derived from T_1 _mapping may be particularly useful for infarct size and area at risk calculations, because it is inherently insensitive to signal variation due to confounders such as RF excitation and receive inhomogeneities.

## Funding

Ben B. and Iris M. Margolis Foundation.
